# *Haemoproteus ilanpapernai* n. sp. (Apicomplexa, Haemoproteidae) in *Strix seloputo* from Singapore: morphological description and reassignment of molecular data

**DOI:** 10.1051/parasite/2014018

**Published:** 2014-04-24

**Authors:** Grégory Karadjian, Ellen Martinsen, Linda Duval, Jean-Marc Chavatte, Irène Landau

**Affiliations:** 1 UMR 7245 MCAM MNHN CNRS, Muséum National d’Histoire Naturelle 61 rue Buffon CP 52 75231 Paris Cedex 05 France; 2 Smithsonian Conservation Biology Institute, Center for Conservation and Evolutionary Genetics, National Zoological Park PO Box 37012 MRC 5503 Washington DC 20013-7012 USA; 3 Malaria Reference Centre – National Public Health Laboratory, Ministry of Health, 9 Hospital Drive, Block C, #04-01, Sing Health Research Facilities Singapore 169612

**Keywords:** *Haemoproteus ilanpapernai* n. sp., *Strix seloputo*, Singapore, cyt b, co1

## Abstract

*Haemoproteus ilanpapernai* Karadjian and Landau n. sp. from the Spotted Wood Owl, *Strix seloputo,* in Singapore is described from material from Ilan Paperna’s collection of slides. The species was previously identified as *Haemoproteus syrnii* (Mayer, 1910). However, comparisons between the material from *Strix seloputo* and our own material from *Strix aluco,* the type host of *H. syrnii,* revealed morphological and molecular differences. *H. ilanpapernai* n. sp. differs morphologically from *H. syrnii* by the much smaller size of the gametocytes, the different position of the mature gametocytes in the erythrocyte (apical, subapical, or lateral in *H. ilanpapernai* vs. always lateral in *H. syrnii*), the effect on the erythrocyte nucleus (frequently tilted in *H. ilanpapernai* but not displaced laterally vs. straight and displaced laterally in *H. syrnii*) and characters of the pigment (aggregated in the gametocytes of *H. ilanpapernai* vs. dispersed in *H. syrnii*). A molecular analysis showed that the two species differ by 2.9% at the cyt b and 3.1% at the COI genes.

## Abstract (fr)

*Haemoproteus ilanpapernai* Karadjian et Landau n. sp. chez la chouette des pagodes, *Strix seloputo*, de Singapour, est décrit à partir des lames de la collection d’Ilan Paperna. L’espèce avait été précédemment identifiée en tant que *Haemoproteus syrnii* (Mayer, 1910). Néanmoins, les comparaisons entre le matériel provenant de *Strix seloputo* et notre propre matériel provenant de *Strix aluco*, l’hôte type de *H. syrnii*, ont révélé des différences morphologiques et moléculaires. *H. ilanpapernai* n. sp. diffère morphologiquement de *H. syrnii* par une taille du gamétocyte plus petite, les positions différentes du gamétocyte mature dans l’érythrocyte (position apicale, subapicale ou latérale chez *H. ilanpapernai* vs. toujours latérale chez *H. syrnii*), l’effet sur le noyau du globule rouge (fréquemment pivoté et non déplacé latéralement chez *H. ilanpapernai* vs. non pivoté mais déplacé latéralement chez *H. syrnii*) et les caractères du pigment (agrégés dans les gamétocytes de H*. ilanpapernai* vs. dispersés dans les gamétocytes de *H. syrnii*). Une analyse moléculaire a montré que les deux espèces diffèrent de 2,9 % sur le gène cyt b et de 3,1 % sur le gène COI.

## Introduction

Several *Haemoproteus* have been reported in Strigidae from different localities in South-East Asia but only two were described morphologically by Ilan Paperna [1]: *H. noctuae* Celli and San Felice, 1891 [2] in the Brown Hawk-Owl *Ninox scutulata* (Raffles, 1822), and *H.* cf. *noctuae* in *Glaucidium brodiei* (Burton, 1836). A third *Haemoproteus* species, identified as *H. syrnii* (Mayer, 1910) [3], was also found in one *N. scutulata* and in a *Strix seloputo* Horsfield, 1821 [1].

Using a blood sample collected by Paperna from a *S. seloputo* in Singapore, Martinsen et al. [4] published the first gene sequences from this parasite. The corresponding material from Ilan Paperna’s collection was later deposited in the Muséum National d’Histoire Naturelle, Paris. After study of the corresponding blood samples we were able to describe the present species and differentiate it from *H. syrnii*.

We found that the morphology of the parasites of *Strix* from Singapore corresponded neither to the original description by Mayer (1910) of *H. syrnii* in *Strix aluco* (Linnaeus, 1758) from Germany and Austria [3] nor to the morphology of *H. syrnii* in *S. aluco* from different regions in France [5]. Furthermore, the mitochondrial sequences obtained by Martinsen et al [4] from the cyt b and COI genes of the parasites from *S. seloputo* differ significantly from those we obtained from the parasites of *S. aluco* in France [5]. We were therefore dealing with two different species.

## Material and methods

### Biological material

According to Paperna et al. [1], the birds were collected with mist nets in Singapore, in two forests in the central water catchment area (Nee Soon and MacRitchie 1° 22′ N, 103° 48′ E [6])”.

Two raptor species were found infected with the parasite identified at the time as *H. syrnii*: *N. scutulata*, Owl 1, June 2001, and *S. seloputo*, Owl 3, 2003. Owl 1 (*Ninox scutulata*) was also infected by *Plasmodium ninoxi* [1].

The material of the present description is based on slides from *S. seloputo* (Owl 3) sampled on the same day and harboring a pure infection. It comprises blood smears and a blood spot from this bird which were sent to Martinsen for molecular analysis [4]. There is no indication of the number of birds examined in Singapore. Morphological comparisons with *H. syrnii* were made with blood smears of seven adult *S. aluco* from the Cévennes, Hérault (France), and molecular characterization was performed on two blood samples (one EDTA tube and one blood spot) which harbored single infections with *H. syrnii*.

### Methods

All blood smears were fixed using absolute methanol prior to Giemsa staining (10% in phosphate-buffered solution, pH = 7.4) for 1 h. They were then covered by a cover slip mounted with Eukitt^®^ resin before examination under oil immersion, as previously described [5].

The DNA extractions and PCR protocols have previously been described [4, 5]. A p-distance analysis was performed on the common gene portions (360 bp for cyt b and 945 bp for COI).

### Photographs and measurements

The blood smears were examined with an Olympus BX63 microscope and the microphotographs performed with an Olympus DP72 camera. Measurements were performed on the microphotographs using the cellSens Dimension 1.9 software.

### Statistical analysis

Kolmogorov-Smirnov [7] and Shapiro-Milk [8] normality tests were performed at first. The values of the parasites’ sizes do not follow a normal distribution and Mann and Whitney [9] tests were performed to analyze the differences between the two parasite species’ length and width. The values of the red blood cells’ sizes follow a normal distribution and one-way ANOVA tests were performed to measure the length and the width of non-parasitized red blood cells and cells parasitized by male and female gametocytes. Data analyses were performed with the GraphPad Prism 5 software.

## *Haemoproteus ilanpapernai* Karadjian and Landau n. sp.

urn:lsid:zoobank.org:act:17FC0A4D-DE7E-47E7-9A3E-0FA1DF49FBA6

Type host: *Strix seloputo* Horsfield, 1821.

Type locality: Singapore.

Collector and date: Ilan Paperna, 2001–2003.

Etymology: named after the late Ilan Paperna.

Other host: *Ninox scutulata* (Raffles, 1822).

Type material: 8 blood films from a *Strix seloputo* deposited in the collections of the Muséum National d’Histoire Naturelle, Paris (MNHN 176BF, PXIV58- 63).

Authority: The authors of the new taxon are different from the authors of this paper; Article 50.1 and Recommendation 50A of the International Code of Zoological Nomenclature [10].

### *Description* (Figs. 1–16, Table 1)

**Figures 1–16:**
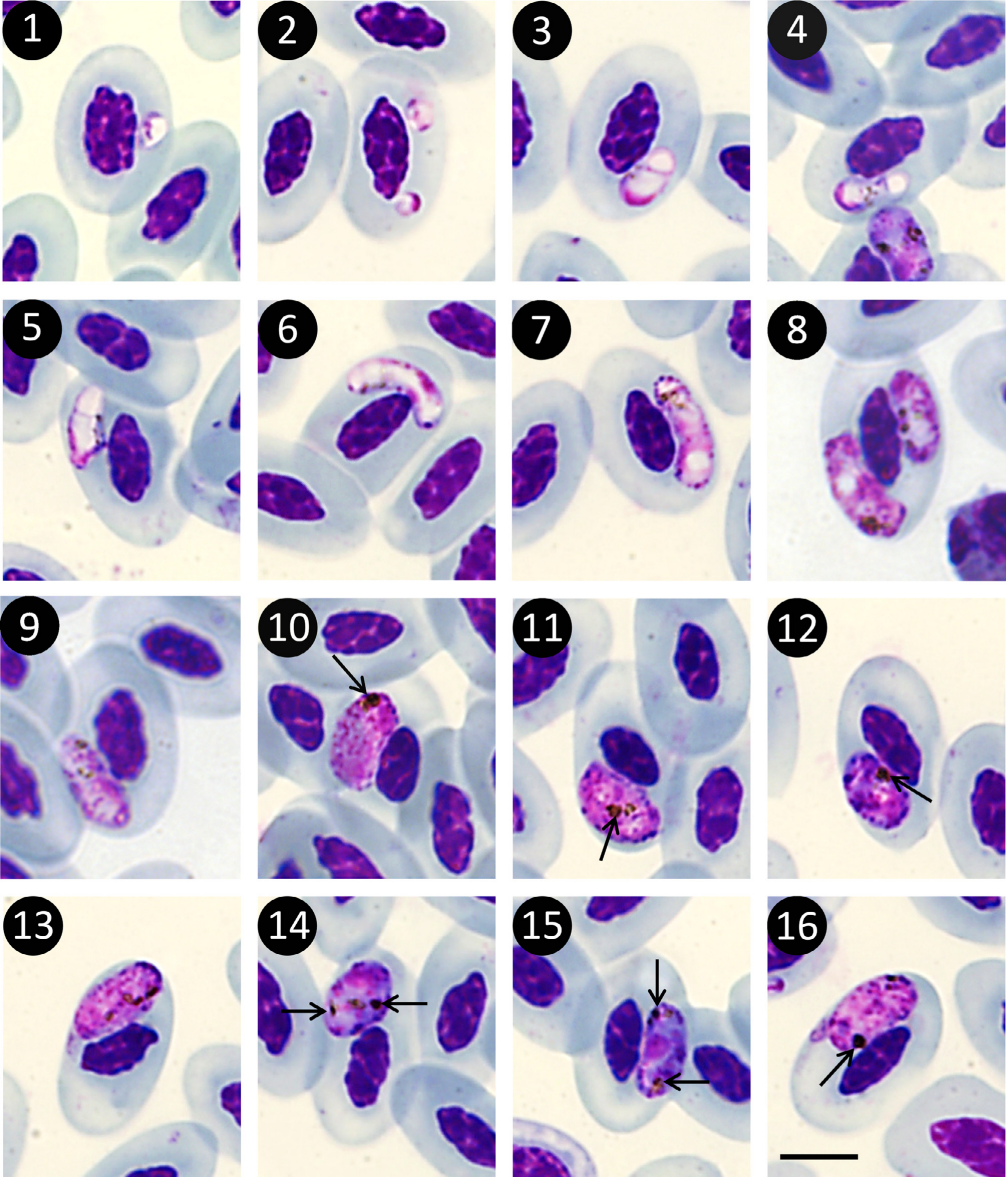
Microphotographs of gametocytes of *Haemoproteus ilanpapernai* Karadjian & Landau n. sp. in the blood of *Strix seloputo*. 1–5: Young gametocytes; 6 and 7: immature gametocytes; 8 and 9 : nearly mature gametocytes; 10 and 16: microgametocytes with agglomerated pigment (arrows); 11 and 13: microgametocytes with the erythrocyte nucleus tilted; 12: macrogametocyte with the erythrocyte nucleus tilted; 14 and 15: macrogametocytes with aggregation of pigment (arrows). Giemsa staining. Scale bar = 5 μm.

Young gametocytes (Figs. 1–5) at first round or oval with the nucleus at one end and a large intra-cytoplasmic vacuole (Figs. 3–5); then elongated along the RBC nucleus, parasite nucleus median, and both extremities containing large white vacuoles (Figs. 6–9). Small dark brown granules and fine rods of dark brown pigment scattered in the cytoplasm. Gametocytes along the erythrocyte nucleus, sometimes at its end (Fig. 6). Volutin granules at the periphery, round and well individualized (Figs. 8 and 9).

**Table 1.** Size of parasites and red blood cells.

	Uninf RBC	Micro RBC	Macro RBC	Micro	Macro
Length (µm)	14.83 ± 0.50	14.54 ± 0.65	14.55 ± 0.62	7.85 ± 0.70	7.08 ± 0.61
Width (µm)	8.39 ± 0.52	8.47 ± 0.47	8.39 ± 0.55	3.98 ± 0.50	3.60 ± 0.46

Microgametocytes (micro), macrogametocytes (macro), uninfected red blood cells (uninf RBC), red blood cells harboring microgametocytes (micro RBC), macrogametocytes (macro RBC). Results are expressed as mean length and width ± SEM, *n* = 30. The sizes of the microgametocytes and the macrogametocytes were analyzed by the Mann-Whitney test; mature microgametocytes are significantly larger than macrogametocytes (length, *p* value < 0.0001; width, *p* value < 0.001).The sizes of RBC were tested by a one-way analysis of variance and showed no significant difference (length, *p* value = 0.11; width, *p* value = 0.79).

Mature gametocytes, 67% of the total number of gametocytes, compact, ellipsoid, or rounded, and located near the erythrocyte’s nucleus, touch the nucleus without being closely adpressed to it (Figs. 10–16). Microgametocyte nucleus, diffuse with few aggregations of chromatin. Macrogametocyte nucleus rounded and well limited. Disappearance of the large vacuoles of the immature stages; numerous small volutin grains scattered in the cytoplasm, particularly at the periphery (Figs. 10–16). Dark brown pigment of the microgametocytes aggregated, forming a dense mass (Figs. 10, 11, 16), pigment of the macrogametocytes more dispersed (Figs. 12, 14, 15). Mature microgametocytes significantly larger (7.85 ± 0.70 μm × 3.98 ± 0.50 μm) than macrogametocytes (7.08 ± 0.61 μm × 3.60 ± 0.46 μm) (Mann-Whitney test, respectively, *p* < 0.0001 and *p* < 0.001, *n* = 30) (Table 1). Length/width ratio identical in both sexes.

Characteristics of the parasite: no particular position inside the erythrocyte. May be found in an apical, latero-apical, or lateral position. Host cell not hypertrophied (Table 1). Erythrocyte nucleus not displaced laterally and on the same level as the parasite. Nucleus of the erythrocyte sometimes tilted, obliquely, or perpendicularly to the blood cell axis, according to the position of the gametocyte (Figs. 11–13).

### Molecular data

The sequences from cyt b and COI of *H. ilanpapernai* n. sp. previously associated with *H. syrnii* [4] are available in GenBank (DQ451424, EU254591). Our sequences of *H. syrnii* are deposited in GenBank as KF279522 and KF279523. Genetic distance analysis (*p*-distance) shows that the two species of *Haemoproteus* differ by 2.9% at the cyt b gene and 3.1% at the COI gene [5].

### Differential diagnosis

*H. ilanpapernai* can be differentiated from *H. syrnii* by its smaller length (7.8 μm vs*.* 16.3 μm). The two species also differ by a number of other morphological characters. In *H. ilanpapernai* n. sp., the shape is ellipsoid or rounded, the position inside the erythrocyte is variable, the erythrocyte nucleus is central and frequently tilted, and the pigment of the mature gametocyte is rough and agglomerated. In contrast, the gametocytes of *H. syrnii* have an elongated shape, a lateral position along the erythrocyte nucleus, they displace the erythrocyte nucleus laterally, and they have dispersed pigment.

*H. ilanpapernai* n. sp. differs from the two other species described by Paperna in the Strigidae of Singapore: the gametocytes of *H. noctuae* in *Ninox* are much larger than those of *H. ilanpapernai* n. sp., sometimes completely surround the host cell’s nucleus and are devoid of volutin granules; the gametocytes of *H. cf noctuae* from *Glaucidium* contain volutin granules but are much larger than those of *H. ilanpapernai* n. sp. They are amoeboid with conspicuous cytoplasmic projections, while *H. ilanpapernai* n. sp. is a small parasite with an even contour.

## Discussion

Paperna et al. [1], noticing the small size of the gametocytes, thought that only immature parasites were present in the blood smears of the owl. In fact, the majority of gametocytes are fully differentiated into mature micro- and macrogametocytes. Since, at that time, no sequence of identified parasites from *Strix* was available in GenBank, the cyt b and COI sequences from *S. seloputo* were therefore assigned to *H. syrnii.* Two other non-identified cyt b sequences from *Haemoproteus* parasites of *Strix varia* (Barton, 1799) from Austria [11, 12] can be retrieved from GenBank and show 0.5% differences with *H. syrnii*. They are probably another haplotype of *H. syrnii*.

In view of the important morphological differences between *H. ilanpapernai* n. sp. and *H. syrnii,* we consider that these two parasites should be considered as two different species. The cyt b and COI sequences of *H. ilanpapernai* n. sp. show differences of, respectively, 2.9% and 3.1% with *H. syrnii*, which confirms the morphological analysis.

The sequences previously deposited in GenBank and assigned to *H. syrnii* [4] should be reassigned to *H. ilanpapernai* n. sp. and the geographical origin of the samples stated mistakenly as Israel should be changed to Singapore.

The number of sequences of bird *Haemoproteus* deposited in databases is increasing and their specific identification is very often a problem, as pointed out by Valkiūnas et al. [13] and Karadjian et al. [5]. This problem arises mainly from the diversity of parasite species present in a single host. In the case of *H. ilanpapernai*, we are as confident as possible that the owl harbored a single species of *Haemoproteus*.
